# Primary osteosarcoma of the breast during lactation: a case report and literature review

**DOI:** 10.3389/fonc.2024.1362024

**Published:** 2024-11-06

**Authors:** Huifang Zhuo, Yubei Duan, Shuangshuang Dong, Jun Zhang, Zijing Wang, Lian Meng, Chenyan Wang, Man Li, Xiaotian Yang, Ning Wang, Jianming Hu

**Affiliations:** ^1^ Department of Pathology and Key Laboratory for Xinjiang Endemic and Ethnic Diseases (Ministry of Education)/Department of Pathology, the First Affiliated Hospital, Shihezi University School of Medicine, Shihezi, Xinjiang, China; ^2^ National Health Commission (NHC) Key Laboratory of Prevention and Treatment of Central Asia High Incidence Diseases (First Affiliated Hospital, School of Medicine, Shihezi University), Shihezi, Xinjiang, China; ^3^ Department of Pathology, Northern Jiangsu People's Hospital Affiliated to Yangzhou University/Clinical Medical College, Yangzhou, China; ^4^ Department of Function, the First Affiliated Hospital, Shihezi University School of Medicine, Shihezi, Xinjiang, China; ^5^ Department of Internal Medicine, Fourth People's Hospital, Urumqi, Xinjiang, China

**Keywords:** breast malignancy, primary osteosarcoma of the breast, lactation, pathological diagnosis, case report

## Abstract

Primary osteosarcoma of the breast (POB) is an aggressive and exceedingly rare tumor, and cases with onset during lactation are extremely rare. The exact mechanism of POB development remains unclear. They may originate from totipotent mesenchymal cells in the breast stroma or may be the result of neoplastic transformation of prior breast lesions. Here, we present the case of a 40-year-old Chinese woman who was found with a palpable tumor measuring approximately 3 cm in diameter in her right breast while breastfeeding 4 months post-partum. The lactating woman was misdiagnosed with lactational mastitis during her initial visit to a community hospital. However, due to negative bacterial cultures and ineffective anti-infective treatment, later on the patient was taken to a more advanced hospital where a tissue biopsy was taken. The superior hospital considered that it might be a malignant tumor and performed local excision of the breast mass, leading to a final pathological diagnosis of POB. This is the first reported case of primary osteosarcoma during breastfeeding. We hope that this case report will improve readers’ understanding of the diagnosis and differential diagnosis of POB, especially for patients with atypical clinical symptoms and imaging findings, which should not be taken lightly.

## 1 Introduction

Primary osteosarcoma of the breast (POB) is a rare form of extraskeletal osteosarcoma originating from soft tissue. Its incidence is less than 1% among malignant breast tumors, accounting for approximately 12.5% of breast sarcomas (1–3). The exact mechanism of POB development is still unclear. Studies have shown that it may arise from neoplastic transformations of prior breast lesions such as fibroadenomas, phyllodes tumors, intraductal papillomas, and from totipotent mesenchymal cells in the breast stroma (4, 5). One possible mechanism is that, under the stimulation of radiotherapy, chronic lymphedema, trauma, or other stimuli, epithelial cells undergo mesenchymal transformation, or ossification, and further malignant transformation into osteosarcoma (6). Compared with breast cancer, POB carries a poorer prognosis, with early tumor recurrence tending to be hematogenous rather than lymphatic and most commonly metastasizing to the lungs (1, 7, 8). Presently, most reports of POB are individual cases, and cases diagnosed during lactation have not been reported. Here, we present the case of a 40-year-old lactating female diagnosed with POB of the right breast. We report the clinicopathological manifestations in this patient and discuss its diagnosis and treatment within the context of a review of relevant literature.

## 2 Case report

**Figure 1:**
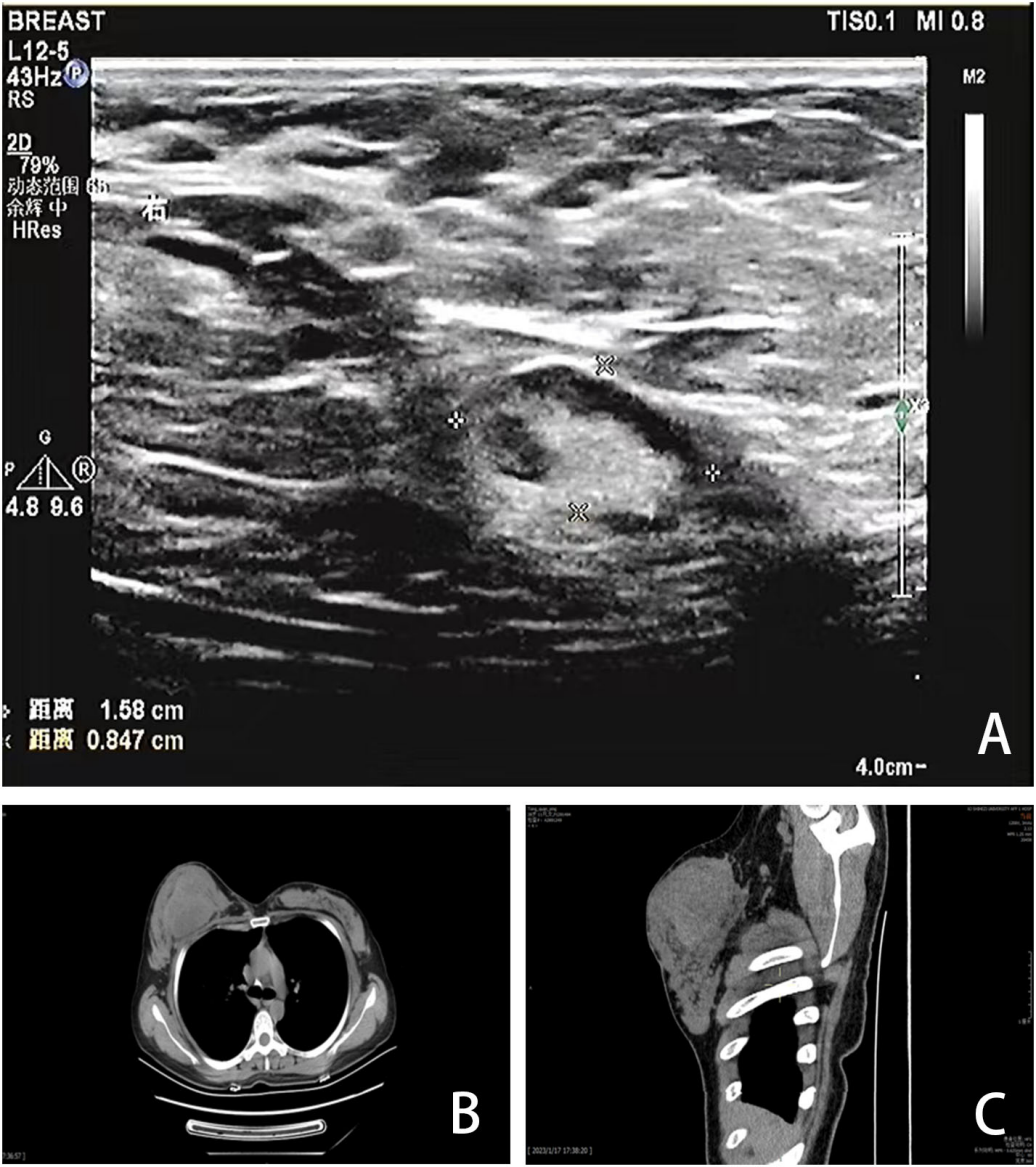
Imaging findings. **(A)** CDUS showed a heterogeneous mixed echo area of 9.7×6.0×10cm in the right breast. **(B, C)** CT showed a round mass in the right breast with patchy low-density patches and septations, with a diameter of 7.1×5.2cm.

A 40-year-old Chinese woman, who had been breastfeeding for four months, sought medical attention due to a lump in her right breast. She did not experience any symptoms such as localized pain, skin redness, bleeding, or leakage. She was previously in good health, with no family history of genetic diseases or breast-related diseases and had never undergone breast ultrasound or mammography. She had been pregnant three times and breastfed three healthy children. Upon admission, a physical examination revealed symmetrical thoracic cage and bilateral breasts, with no redness or superficial varicose veins on the surface of the skin, no nipple inversion, no “dimple sign”, no “orange peel-like change”, and a 10×10cm mass in the right breast areola area with high tension, hard texture, and slightly higher skin temperature. Initially seeking care at a community hospital that only provides general practitioners with primary care, a color Doppler ultrasound (CDUS) revealed a mixed echo area in the right breast measuring 60×33 mm, suggesting inflammatory changes. She was diagnosed with a breast abscess and prescribed with oral ceftriaxone for eight days following three days of oral penicillin treatment. However, there was no improvement. A month later, she was referred to the First Affiliated Hospital of Shihezi University. The CDUS examination revealed rough echoes in both breasts, with an inhomogeneous mixed echo area approximately 9.7×6.0×10 cm in size in the right breast, exhibiting an unclear boundary and an irregular shape (**Figure 1A**). Color Doppler flow imaging (CDFI) showed blood flow signals therein. The contralateral breast showed no significant space-occupying lesions. Based on these imaging data, the lesions were initially considered to be inflammatory by the imaging physicians at the First Affiliated Hospital. Chest computed tomography (CT) revealed a round mass shadow with patchy low-density patches and septa in the right breast measuring approximately 7.1×5.2 cm in diameter. Apart from the mediastinum and significant arteries, no swollen lymph nodes were observed (**Figures 1B, C**). Under the guidance of ultrasound, the abscess was punctured and the sample was cultured for bacteria, which showed a negative result. Considering the malignant tumor, the surgeon opted for breast lesion resection and pathological biopsy to remove the lesion and simultaneously clarify the diagnosis.

**Figure 2:**
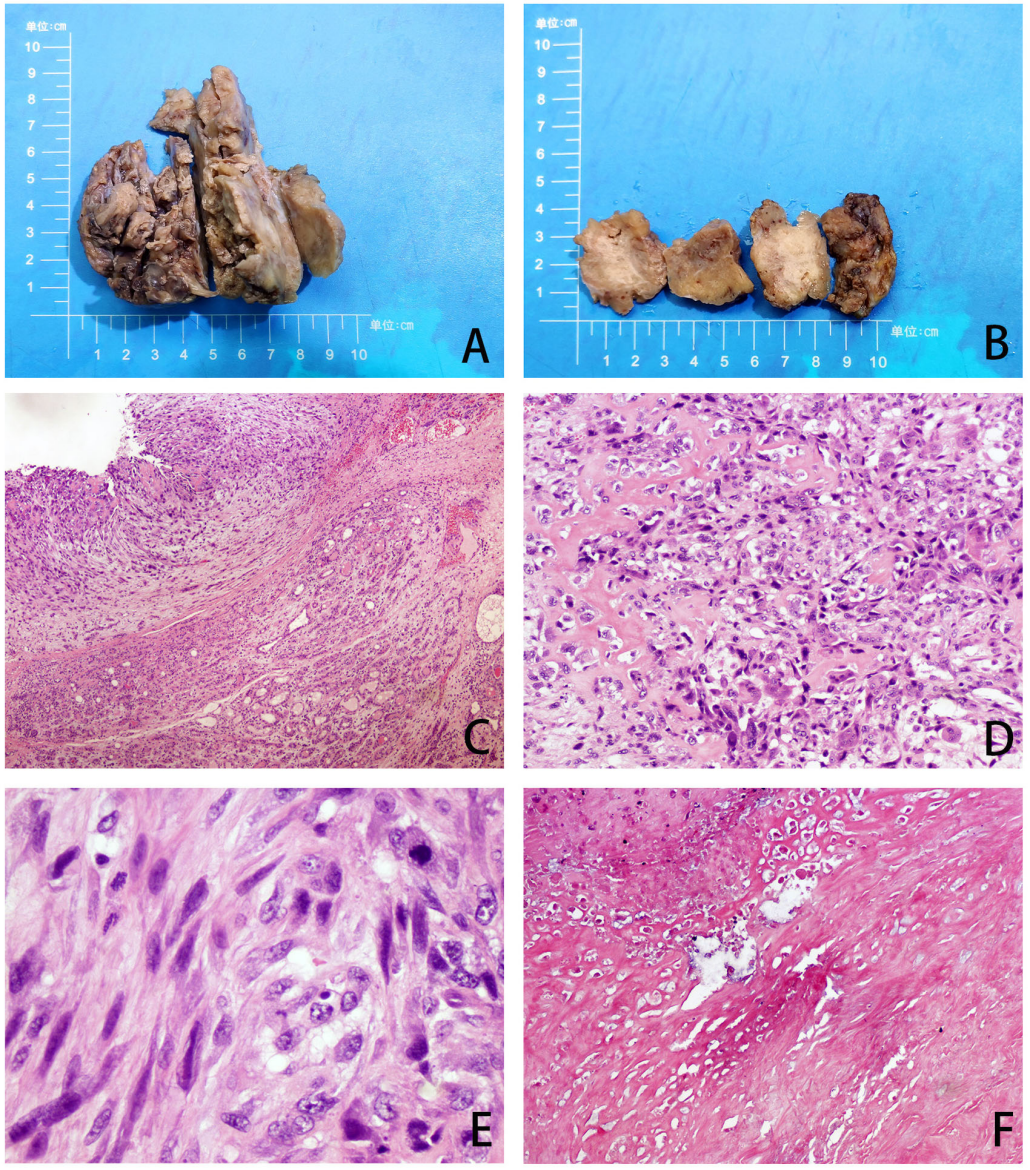
**(A, B)** Gross observation of lesion resection specimens. **(C)** The tumor and adenoma areas of breast tissue were clearly demarcated (H&E, ×40). **(D)** The tumor is spindle-shaped and shows an obvious osteoid matrix, giant cells, and osteogenesi. (H&E, ×100). **(E)** Under high magnification, pathological mitotic images are easily visible (H&E, ×400). **(F)** Extensive necrosis (H&E, ×40).

**Figure 3:**
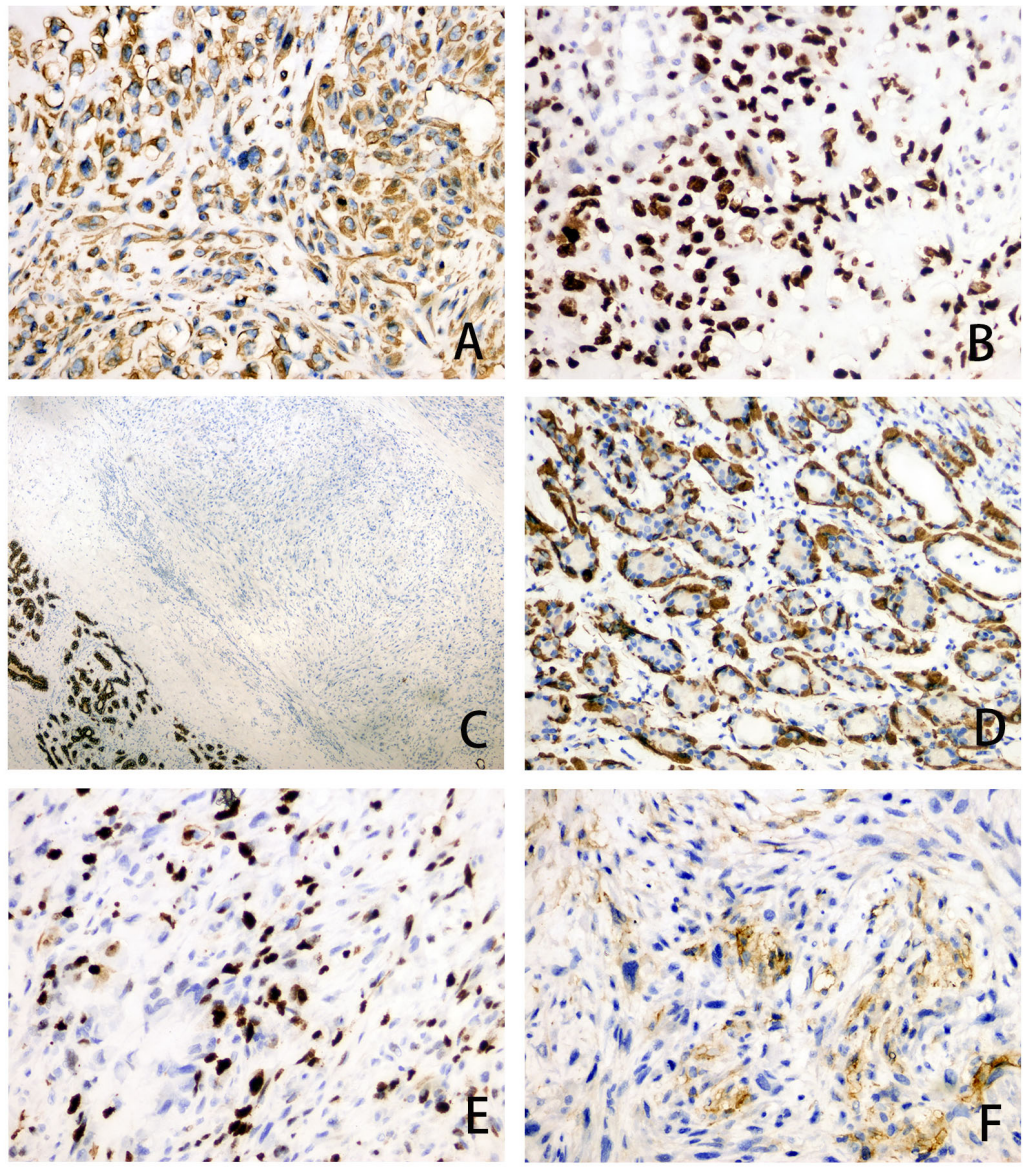
**(A–F)** Immunohistochemical staining of tumor area for Vimentin (magnification, ×200), SATB2 (magnification, ×200), CK5/6 (magnification, ×40), SMA (magnification, ×200), Ki-67 (magnification, ×200) and CD99 (magnification, ×200).

The patient underwent excision of the inflammatory necrotic tissue, resulting in the removal of approximately 8.4×7.5×3.5 cm of non-neoplastic broken tissue (**Figure 2A**). The lesion tissue was partly cystic and partly solid, with a slightly dark red inner cyst wall and surrounding nodules measuring 4.5×3.3×3.2 cm. The nodular section was gray and grayish-yellow, and the texture was hard. Some areas exhibited lustrous surfaces and a mucous-like consistency (**Figure 2B**). The tissue excised from the right breast was subjected to histological examination. Microscopically, the breast tissue showed lobular hyperplastic changes with adjacent vesicles and sarcomatoid changes in the cyst wall. A clear demarcation between tumor and hyperplastic areas was evident (**Figure 2C**). The osteoid matrix, giant cells, and osteogenesis were observed in some areas of the tumor. Tumor cells displayed spindle-shaped or polygonal morphology with atypia (**Figure 2D**). High-power magnification imaging showed spindle cells arranged in bundles, with some exhibiting swirling or random distribution. Cells displayed sparse cytoplasm, unclear boundaries, deep nuclear staining, and round, oval, or short spindle shapes. Vacuolar changes were noted in the spindle cell cytoplasm, along with aberrant pleomorphic tumor cell mitosis and prevalent pathological mitosis (**Figure 2E**). Extensive necrosis was observed in peripheral areas (**Figure 2F**). This pathological form must be differentiated from metaplastic breast carcinoma by osteomatrix differentiation. So, we performed a biopsy of all tumor tissue, but did not find any epithelial component on the histomorphology. We performed further an immunohistochemical examination, revealing that the tumor area was positive for vimentin and SATB2 (**Figures 3A, B**), while CK5/6 and SMA were negative (**Figures 3C, D**). The Ki-67 immunohistochemistry revealed a proliferation index of approximately 55% in breast adenosis adjacent to osteosarcoma. CD99 immunohistochemical staining showed scattered and weakly positive staining (**Figures 3E, F**). Based on the morphological and immunohistochemical findings, no epithelial component was found and we excluded metaplastic breast carcinomas with osteomatrix differentiation. Therefore, the patient was diagnosed with POB. POB is not suitable for breast cancer staging systems because of its sarcomatoid nature. Following the staging classification for osteosarcoma established by the American Joint Committee on Cancer (AJCC), the patient was in stages T_2_N_0_M_0_. The patient was in stage G3 according to the French Federation of Cancer Centers Sarcoma Organization (FNCLCC) staging system for pathological grading, and the patient was in stage IIA according to the combined AJCC and FNCLCC staging system.

The patient underwent modified radical mastectomy on the right breast after diagnosis of breast osteosarcoma. No residual tumor tissue was found at any cutting edge of the breast. Postoperative chemotherapy with ifosfamide and adriamycin was administered in 21 days per cycle, spread over 6 months. The patient had nausea, vomiting, and loss of appetite during chemotherapy, but all were within the patient’s acceptable range. We established a follow-up plan that included outpatient visits every three months in the first year, every six months in the second year, and then annually or at longer intervals as clinically indicated. Each follow-up visit included a complete physical examination, a bone scan, chest imaging (mammography and chest CT), and functional scoring. After 12 months of follow-up, the patient’s overall condition was good, and no metastasis or recurrence was found on imaging. The patient was satisfied with the outcome of the treatment, and there were no obvious complications from the treatment.

## 3 Discussion

POB is a rare and poorly differentiated tumor (9). The etiology of POB remains unclear, with reports suggesting associations with a history of burns, trauma, and even foreign bodies (2, 9, 10) and even radiation therapy (11, 12). Most patients have a several-year history of breast masses, with sudden short-term enlargement (2). In this case, the patient experienced the rapid formation of round, hard, heterogeneous, and well-defined masses during lactation. Clinically, lactation period is a high-risk period for purulent mastitis, which may also be a predisposing factor. Radiological features of POB often resemble those of benign tumors, typically showing hypoechoic diffuse coarse calcification, rich blood flow, and clear boundaries (13). Histopathology serves as the primary basis for distinguishing between benign and malignant tumors. Imaging tests such as ultrasound and CT have limited utility in differentiating purulent mastitis and POB (14).

For an accurate diagnosis of POB, a comprehensive evaluation of clinical, radiological, and pathological features is necessary. In this case report, the patient was misdiagnosed primarily because the initial general practitioner at a community hospital considered it a typical lactating mastitis based on their experience and after CDUS findings. Other diseases were not considered, mainly because the community physician very commonly encountered patients with breast nodules who had lactational mastitis. In addition, community hospitals lack breast specialists to provide counseling or use advanced diagnostic techniques to help detect breast tumors. It was not until the patient was transferred to the First Affiliated Hospital that the bacterial culture was performed, and the results were negative. Based on the negative bacterial culture results, the ineffectiveness of previous antibiotic therapy, and the rapid increase in the mass diameter from 3 cm to 10 cm in one month, the breast specialists at the First Affiliated Hospital felt that a malignant tumor needed to be considered. As a result, the patient underwent a pathological biopsy and immunohistochemistry, which revealed a typical malignant tumor pattern, leading to the diagnosis of POB after ruling out metastatic lesions.

POB shares histopathological similarities with extraskeletal osteosarcoma (ESOS) in other areas of the body, and POB must be distinguished from epithelial-derived metaplastic carcinomas of epithelial origin. Partial metaplastic carcinoma has few epithelial components and presents with atypical osteoid or chondroid tissues that are easily misdiagnosed as osteosarcomas (15). Collecting sufficient samples for the detection of malignant epithelial components is the primary method for differential diagnosis. The non-epithelial characteristics of this tumor were demonstrated by the immunohistochemical results, which were negative for AE1/3 and CK5/6 and positive for vimentin and SATB2. It should also be differentiated from malignant phyllodes tumors, and the key to its differential diagnosis is extensive sampling. Malignant phyllode tumors exhibit prominent stromal atypia and a fibrosarcomatoid appearance, and the intratumoral cystic cavity changes into narrow-branch (16). Benign bone islands coated with osteoblasts may be observed during benign bone tissue metaplasia. Differential diagnosis may involve bone scans and the absence of clinical evidence of extramammary soft tissue or bone osteosarcoma. Detection of neoplastic osteogenesis in breast metastases of osteoblastic osteosarcoma can be challenging, therefore, combining medical history and immunohistochemical staining is essential.

POB presents as an aggressive tumor prone to recurrence and blood system metastasis, characterized by rapid disease progression and a poor prognosis. The 5-year survival rate hovers around 38% (9). According to the literature, the 5-year survival rate of osseous osteosarcoma is reported to be 27.4% for patients with metastases at the initial diagnosis and 70% for patients without metastases (17). Alarmingly, over two-thirds of cases experience recurrence post-local resection, with 11% recurrence even after total mastectomy. In our investigation spanning from 2000 to 2023, we identified 29 patients (**Supplementary Table 1**, **Supplementary Figure 1**). All 29 patients with POB were female, aged between 24 and 96 years, with a median age of 67 years. Tumor diameters ranged from 1.4 to 18.0 cm, with a median diameter of 4.5 cm (2, 4, 7, 9, 18–35). Notably, none of the collected cases reported primary osteosarcoma of the breast during lactation. Our case findings revealed a 1-year survival rate of approximately 77.90% and an expected 3-year survival rate of about 53.68% (**Supplementary Figure 2**). Notably, we observed a correlation between poor prognosis in patients with POB and tumor volume as well as tumor stage (**Supplementary Tables 2**, **3**). When comparing cumulative survival rates across POB stages I to IV, we found the 1-year cumulative survival rate to be the lowest in stage IV cases (**Supplementary Figure 3**). Presently, the standard treatment protocol for POB entails surgical intervention coupled with chemotherapy, primarily employing drugs such as adriamycin (ADM), cisplatin (DDP), and high-dose methotrexate (MTX) (36).

The current targeted therapy for POB is similar to that for common osteosarcoma and focuses on the following targets: The mammalian target of rapamycin (mTOR) protein can hinder the growth of tumors by controlling the cell cycle of tumor cells. Vascular endothelial growth factor (VEGF) can impact the behavior of tumor cells by influencing the development of blood vessels surrounding the tumor. Breast cancer (BRCA) genes can facilitate the repair of DNA damage and influence the advancement of osteosarcoma. Epidermal growth factor receptor (EGFR) may mediate specific pathways to impact tumor cells (37). Recent studies have highlighted the expression of PD-L1 in osteosarcoma cells, with inhibition of PD-1/PD-L1 expression proving beneficial in enhancing cytotoxic T-lymphocyte function (38, 39). Currently, clinical studies on the anti-PD-1 antibody nivolumab and the anti-PD1 antibody pembrolizumab are ongoing. A new targeted agent, glembatumumab vedotin (CDX-011), targets the transmembrane glycoprotein NMB (GPNMB; Osteoactivin) in a preclinical trial, three samples showed sustained complete remission after CDX-011 application (36). Furthermore, kinase ataxia telangiectasia mutated and Rad3-related (ATR) inhibition is a novel approach for osteosarcoma treatment. Osteosarcoma cells utilize an alternative mechanism of telomere elongation to overcome replicative senescence (40), a process commonly associated with ATRX loss. ATR inhibition disrupts this mechanism, leading to chromosome breakage and tumor cell apoptosis. Treatment with the ATR inhibitor VE-821 has shown heightened sensitivity and increased tumor cell death in ALT-positive osteosarcoma cell lines (41).

In the present case, radical mastectomy followed by chemotherapy proved effective using current treatment paradigms. However, vigilant follow-up is necessary to monitor the prognosis closely.

## 4 Conclusion

In conclusion, POB is a rare malignancy, and the exact mechanism of its occurrence remains unclear. It primarily metastasizes through the bloodstream, with the lungs being the most frequently affected site, and surgical resection is by far the most effective treatment modality. In the present case, the patient was initially misdiagnosed as having breast-feeding mastitis, and POB was diagnosed after surgical resection after ineffective anti-infection treatment. This suggests that the definite diagnosis of POB requires attention to immunohistochemistry, adequate pathological sampling, and detailed pathological analysis. This case report offers valuable insights for clinicians in identifying and diagnosing this condition.

## Data Availability

The original contributions presented in the study are included in the article/**Supplementary Material**. Further inquiries can be directed to the corresponding authors.

## Supplementary material

Supplementary Figure 1 Flow chart for screening POB literature.

Supplementary Figure 2 Cumulative survival analysis of 29 patients with POB.

Supplementary Figure 3 The cumulative survival rate of POB patients in stage I, stage II, stage III, and stage IV.
