# Supplementary figures and images for: Primary osteosarcoma of the breast during lactation: a case report and literature review
Source: Front Oncol. 2024 Nov 6;14:1362024. doi: 10.3389/fonc.2024.1362024 (PMC11576453; doi:10.3389/fonc.2024.1362024)

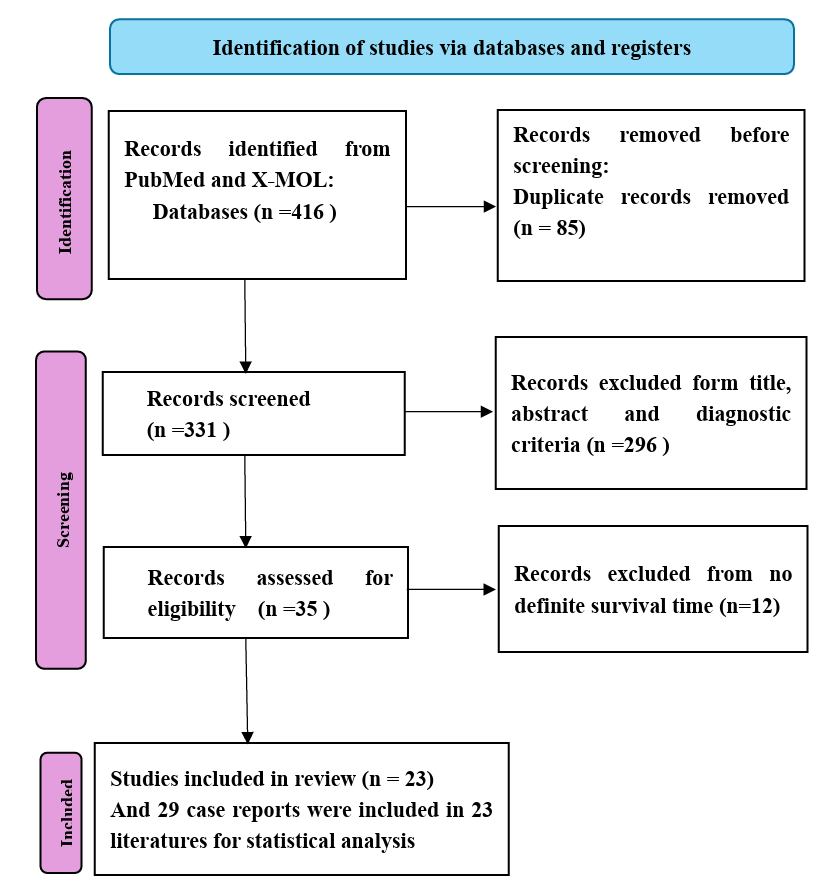

Supplement: Supplementary Figure 1 — Flow chart for screening POB literature. [file Image1.png]

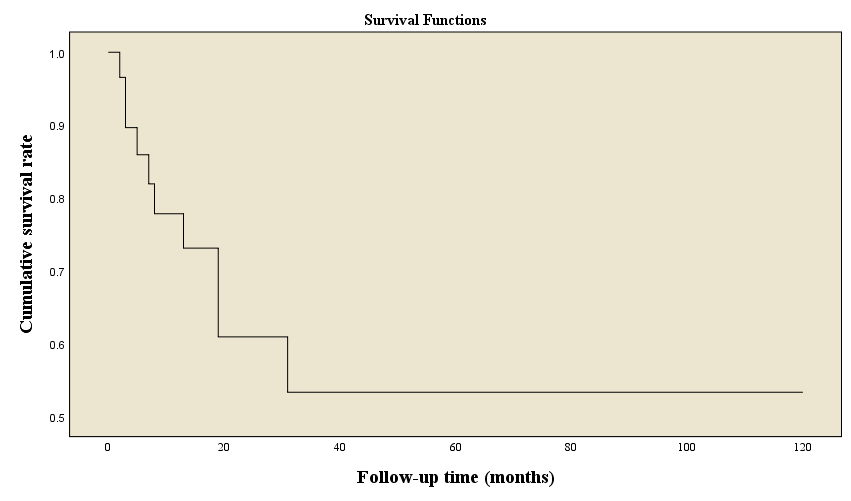

Supplement: Supplementary Figure 2 — Cumulative survival analysis of 29 patients with POB. [file Image2.tiff]

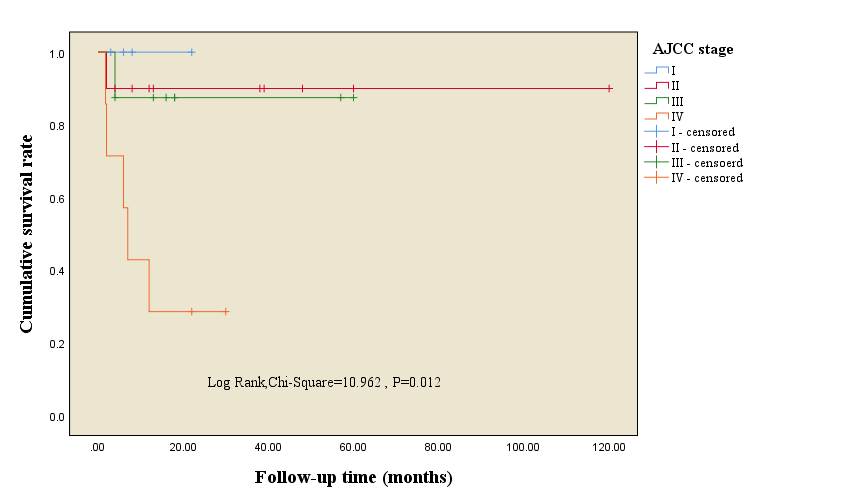

Supplement: Supplementary Figure 3 — The cumulative survival rate of POB patients in stage I, stage II, stage III, and stage IV. [file Image3.tiff]
