# Supplementary material for: Primary osteosarcoma of the breast during lactation: a case report and literature review
Source: Front Oncol. 2024 Nov 6;14:1362024. doi: 10.3389/fonc.2024.1362024 (PMC11576453; doi:10.3389/fonc.2024.1362024)
Supplement: Supplementary file 4 [file Table1.docx]

**Table 1 29 cases of POB were retrieved**

|  | Author | Age(years) | Tumor diameter(cm) | Adjuvant therapy | Follow-up time (months) | AJCC stage |
| --- | --- | --- | --- | --- | --- | --- |
| Case1 | Armita Bahrami et al(2007) | 88 | 18 | N | 16 | III |
| Case2 | Armita Bahrami et al(2007) | 96 | 7.5 | N | 4 | III |
| Case3 | Helen Bartlett (2019) | 73 | 3.5 | Y | 8 | I |
| Case4 | Arvind Krishnamurthy (2015) | 73 | 6 | N | 4 | II |
| Case5 | Mariusz Szajewski et al(2014) | 67 | 7 | Y | 18 | III |
| Case6 | Seshikanth Middela et al(2011) | 69 | 4 | UK | 2 | II |
| Case7 | Helen Trihia et al(2007) | 76 | 4 | Uk | 30 | IV |
| Case8 | Helen Trihia et al(2007) | 55 | 3 | Uk | 38 | II |
| Case9 | Helen Trihia et al(2007) | 48 | 4.5 | Uk | 22 | I |
| Case10 | Chan Seok Yoon and Sung Soo Kang (2017) | 77 | 3.5 | N | 3 | I |
| Case11 | Julie Crevecoeur et al(2016) | 65 | 2.9 | Y | 1.75 | IV |
| Case12 | Shigeki Murakami et al(2008) | 59 | 3 | N | 60 | II |
| Case13 | Prashant Balwant Kerkar and Garima Daga (2018) | 44 | 14 | Y | 13 | III |
| Case14 | Shike Li et al(2022) | 83 | 8 | N | 4 | III |
| Case15 | Ilona A. Dekkers et al(2019) | 67 | 4 | Y | 12 | II |
| Case16 | Ahmad Al Samaraee et al(2015) | 81 | 3 | Y | 48 | II |
| Case17 | Odd T Brustugun et al(2005) | 47 | 1.4 | Y | 22 | IV |
| Case18 | Odd T Brustugun et al(2005) | 59 | 7 | N | 6 | IV |
| Case19 | Odd T Brustugun et al(2005) | 24 | 3 | N | 120 | II |
| Case20 | Odd T Brustugun et al(2005) | 80 | 5 | N | 18 | III |
| Case21 | Ashwin A Kallianpur et al(2013) | 50 | 13 | Y | 57 | III |
| Case22 | JingJie Zhao et al(2013) | 77 | 7 | N | 60 | III |
| Case23 | Anna Rizzi et al(2013) | 62 | 4.2 | Y | 13 | II |
| Case24 | Kristine S. Burk et al(2018) | 67 | 3.2 | Y | 8 | II |
| Case25 | Evariste Gafumbegete et al(2016) | 63 | 15 | Y | 12 | IV |
| Case26 | Shabuddin Khan et al(2008) | 66 | 6 | Y | 6 | I |
| Case27 | K. Irshad et al(2003) | 77 | 2 | N | 39 | II |
| Case28 | Hirohito Momoi et al(2004) | 58 | 9 | Y | 7 | IV |
| Case29 | Boutayeb Saber et al(2008) | 38 | 8 | Y | 2 | IV |

**Table 2** Univariate analysis of prognostic factors in 29 patients with POB

| ***characteristic*** | | ***status*** | | ***One-year cumulative survival rate(%)*** | ***P*** | ***Wald*** | ***RR*** | ***95% CI for RR*** | |
| --- | --- | --- | --- | --- | --- | --- | --- | --- | --- |
|  |  | ***death***  ***（N=7）*** | ***censor***  ***（N=22）*** |  |  |  |  | ***Lower*** | ***Upper*** |
| **Age** |  |  |  | 0.202 | 1.359 | 0.284 | 0.034 | 2.359 |  |
| >=70 | 1（14.3）* | 10（45.5） | 90.0 |  |  |  |  |  |  |
| <70 | 6（85.7） | 12（54.5） | 65.3 |  |  |  |  |  |  |
| **Tumor diameter** |  |  |  | 0.038※ | 3.504 | 4.179 | 0.935 | 18.679 |  |
| >=8 | 4（57.1） | 3（13.6） | 42.9 |  |  |  |  |  |  |
| <8 | 3（42.9） | 19（86.4） | 85.6 |  |  |  |  |  |  |
| **Adjuvant therapy** |  |  |  | 0.846 | 0.088 | 1.177 | 0.399 | 3.473 |  |
| Y | 4（57.1） | 10（45.5） | 68.2 |  |  |  |  |  |  |
| N | 2（28.6） | 9（40.9） | 77.1 |  |  |  |  |  |  |
| **AJCC stage** |  |  |  | 0.015※ | 5.605 | 3.832 | 1.26 | 11.65 |  |
| I | 0（0.0） | 4（18.2） | 100 |  |  |  |  |  |  |
| II | 1（14.3） | 9（40.9） | 90 |  |  |  |  |  |  |
| III | 1（14.3） | 7（31.8） | 87.5 |  |  |  |  |  |  |
| IV | 5（71.4） | 2（9.1） | 28.6 |  |  |  |  |  |  |

*n(n%); ※ Fisher's exact probability method (P<0.05) was statistically significant.

**Table 3** COX stepwise regression analysis of prognostic factors in 29 patients (Forward:LR)

| ***characteristic*** | ***B*** |  | ***df*** | ***P*** | ***Wald*** | ***RR*** | ***95% CI for RR*** | |
| --- | --- | --- | --- | --- | --- | --- | --- | --- |
|  |  |  |  |  |  |  | *Lower* | *Upper* |
| **AJCC stage** | 1.343 |  | 1 | 0.018 | 5.605 | 3.832 | 1.26 | 11.65 |
